# Post-injection delirium/sedation syndrome in patients with schizophrenia treated with olanzapine long-acting injection, I: analysis of cases

**DOI:** 10.1186/1471-244X-10-43

**Published:** 2010-06-10

**Authors:** Holland C Detke, David P McDonnell, Elizabeth Brunner, Fangyi Zhao, Sebastian Sorsaburu, Victoria J Stefaniak, Sara A Corya

**Affiliations:** 1 Lilly Research Laboratories, Eli Lilly and Company, Lilly Corporate Center, Indianapolis, Indiana, USA

## Abstract

**Background:**

An advance in the treatment of schizophrenia is the development of long-acting intramuscular formulations of antipsychotics, such as olanzapine long-acting injection (LAI). During clinical trials, a post-injection syndrome characterized by signs of delirium and/or excessive sedation was identified in a small percentage of patients following injection with olanzapine LAI.

**Methods:**

Safety data from all completed and ongoing trials of olanzapine LAI were reviewed for possible cases of this post-injection syndrome. Descriptive analyses were conducted to characterize incidence, clinical presentation, and outcome. Regression analyses were conducted to assess possible risk factors.

**Results:**

Based on approximately 45,000 olanzapine LAI injections given to 2054 patients in clinical trials through 14 October 2008, post-injection delirium/sedation syndrome occurred in approximately 0.07% of injections or 1.4% of patients (30 cases in 29 patients). Symptomatology was consistent with olanzapine overdose (e.g., sedation, confusion, slurred speech, altered gait, or unconsciousness). However, no clinically significant decreases in vital signs were observed. Symptom onset ranged from immediate to 3 to 5 hours post injection, with a median onset time of 25 minutes post injection. All patients recovered within 1.5 to 72 hours, and the majority continued to receive further olanzapine LAI injections following the event. No clear risk factors were identified.

**Conclusions:**

Post-injection delirium/sedation syndrome can be readily identified based on symptom presentation, progression, and temporal relationship to the injection, and is consistent with olanzapine overdose following probable accidental intravascular injection of a portion of the olanzapine LAI dose. Although there is no specific antidote for olanzapine overdose, patients can be treated symptomatically as needed. Special precautions include use of proper injection technique and a post-injection observation period.

**Trial Registration:**

ClinicalTrials.gov ID; URL: http://http//www.clinicaltrials.gov/: NCT00094640, NCT00088478, NCT00088491, NCT00088465, and NCT00320489.

## Background

Olanzapine long-acting injection (LAI) is a new depot antipsychotic formulation consisting of a pamoate salt of olanzapine that is administered by deep intramuscular (IM) injection every 2 to 4 weeks. Olanzapine LAI has been found to be effective for the treatment of schizophrenia in both actively psychotic [1] and stable patients [2], with a safety profile generally similar to oral olanzapine [2]. However, during clinical trials, a series of cases was identified in which a cluster of adverse events characterized by post-injection delirium and/or excessive sedation was observed [3,4]. These events are believed to be associated with accidental intravascular entry of a portion of the dose, most likely following vessel injury during the injection process [5].

Accidental intravascular injection is a known risk for all intramuscularly injected products and is typically reflected in label warnings. One product with a well-documented example of a post-injection syndrome following accidental intravascular injection is penicillin procaine G [6,7]. When injected intravascularly, the salt formulation dissociates into its penicillin and procaine components, resulting in procaine toxicity, which produces a clear symptomatic presentation known as Hoigne's syndrome. Other intramuscularly injected products that can result in noticeable symptoms following accidental intravascular injection include other long-acting penicillins [8-11], various anesthetic agents used during dental procedures (e.g., Septocaine [12]), as well as promethazine [13], barbiturates and benzodiazepines [14].

With regard to injectable antipsychotics, all advise in their labels against intravascular injection. However, the types of symptoms that might occur or even whether any identifiable symptoms would occur at all, would depend on the formulation (e.g., oil-based, salt-based, microsphere-based) and inherent safety profile of the medication being injected. For long-acting risperidone, for example, rare cases of an embolic-type reaction have been reported with the microsphere formulation. There is recent evidence that a patient with a cardiac malformation (f. ovale) who experienced an accidental intravascular injection of long-acting risperidone developed retinal artery occlusion resulting in persistent blurred vision and superior field deficit in the right eye. Tang and Weiter [15] speculate that the microsphere embolized from the site of injection through the patient's foramen ovale to the right fundus. For haloperidol decanoate and other oil-based typical antipsychotic depot formulations, no specific instances of inadvertent intravascular injection can be found in the literature.

Olanzapine LAI, as a salt-based formulation, may carry risk for a post-injection syndrome as a result of the greater solubility of the salt in blood than in muscle tissue [5]. Moreover, because of the specific adverse-event profile that accompanies the olanzapine molecule, excessive amounts of olanzapine entering the blood stream can result in noticeable symptoms consistent with olanzapine intoxication, particularly excessive sedation (which could include coma) and/or delirium. Because of the possibility of such an occurrence following injection with olanzapine LAI, it is important for clinicians to have a clear understanding of the exact nature of these post-injection syndrome events, including what signs and symptoms to look for in their patients during what time frame, and also what to expect in terms of clinical progression, management, and outcomes following the development of such an event. Termed "post-injection delirium/sedation syndrome" (PDSS) but alternately sometimes referred to as "post-injection syndrome," the first 30 cases identified are presented here along with analyses examining the characteristics, timing, and outcomes of these events as well as an analysis of various patient and injection variables in order to determine whether there are any risk factors which might be used to predict the occurrence of PDSS events.

## Methods

This case analysis was based on all 8 olanzapine LAI clinical trials that were conducted in patients between August 2000 and October 2008. These included a single-dose pharmacokinetic study (n = 134), a 2-month pharmacokinetic study (N = 9), a 6-month pharmacokinetic study (N = 282) [16], a receptor occupancy study (N = 14) [17], an 8-week randomized, placebo-controlled acute efficacy study (olanzapine LAI n = 304) [1], a 24-week randomized, oral olanzapine-controlled maintenance study (olanzapine LAI n = 743) [2], an ongoing 2-year randomized, oral olanzapine-controlled open-label effectiveness study (olanzapine LAI n = 264) [18], and an ongoing 6-year open-label extension study (N = 931) [19]. All patients had a DSM-IV or DSM-IV-TR diagnosis of schizophrenia (n = 2026) or schizoaffective disorder (n = 28) and were between the ages of 18 and 75. Exclusion criteria included significant suicidal or homicidal risk; pregnancy or breastfeeding; acute, serious, or unstable medical conditions; or substance dependency (except nicotine or caffeine) within the past month. All study protocols were approved by institutional review boards at each site. After receiving a complete description of the study, all patients and/or their authorized legal representatives provided written informed consent before participation.

### Olanzapine LAI injection procedures

Patients received their injections after completion of all efficacy and safety assessments at that visit. Injections were generally administered into alternating sides of the buttocks from visit to visit, and administrators were advised not to massage the injection area after injection. Injections were administered using a 19-gauge 1.5-inch (or 35-mm) needle; a 2-inch (or 50-mm) needle could be used for obese patients. Doses ranged from 45 to 405 mg olanzapine pamoate, and injection intervals could be 2, 3, or 4 weeks, depending on the specific study. Injections given at 2-week intervals could not exceed a dose of 300 mg.

Before the PDSS phenomenon was discovered, patients were observed for 10 to 20 minutes following the injection before being released. Based on a review of cases, all ongoing clinical trials were amended in May 2006 to include a 45-minute post-injection observation period and then amended again in August 2006 to include a 3-hour post-injection observation period.

### Variables assessed

In addition to the standard safety assessments collected for all patients (e.g., physical examination, weight, vital signs, reporting of adverse events, laboratory analytes), patients who experienced a PDSS event received other non-scheduled assessments at the discretion of the investigator and/or the treating hospital. These could include sampling of olanzapine plasma concentrations, urine toxicology, and other diagnostic procedures.

### Statistical methods

All analyses were performed on an intent-to-treat basis using SAS version 8.2 (SAS Institute, Inc., Cary, N.C.). Boxplots were created in SigmaPlot version 11 using the Cleveland method of percentile calculation. Unless otherwise specified, summary statistics provided are based on the 30 PDSS events identified as of October 2008. Analyses requiring a locked database relied upon the most recent interim datalock of the ongoing studies, which occurred on 30 April 2008, and which includes 29 PDSS events.

For the purpose of analyses, time of onset of a PDSS event was defined as the time at which the patient first experienced noticeable symptoms and/or the time at which the patient was first witnessed to be experiencing such symptoms. Time of incapacitation was defined as the time at which the patient developed symptoms with the potential to interfere with the patient's ability to seek assistance (e.g., confusion/disorientation, significant ataxia, or severe sedation). Time of hospitalization was defined as the time at which the patient was sent to the emergency department or hospital; if this time was unavailable, time of admission to the emergency department or hospital was used instead.

A logistic regression analysis was performed on the locked database to identify potential risk factors for a PDSS event. Variables included baseline age, gender, race (dichotomized as Caucasian or non-Caucasian), geographic region (dichotomized as United States or outside the United States), dose, number of previous olanzapine LAI injections, and body mass index (BMI) for each patient who experienced a PDSS event and for all who did not experience such an event. A forward stepwise procedure was used to fit the model. The p-value criteria for explanatory variables entering the model and staying in the model were set to 0.20. Risk level was evaluated using odds ratios with 95% confidence intervals (CI).

Concomitant medication use for all patients was assessed up to the time of database lock to determine whether any specific medications or classes of medications posed an increased risk for PDSS. Based on the list of concomitant medications used by PDSS patients at the time of the event, medication classes assessed were anticholinergics, antidepressants (all), selective serotonin reuptake inhibitors (SSRIs), tricyclic antidepressants, anticoagulants, nonsteroidal anti-inflammatory drugs (NSAIDs), angiotensin-converting enzyme (ACE) inhibitors, calcium channel blockers, glucose-lowering drugs, and benzodiazepines/hypnotics. Incidence of concomitant use in patients who experienced PDSS versus patients who did not was compared using Fisher's exact test with an alpha of 0.05.

Potential long-term outcomes associated with PDSS events were assessed by reviewing all spontaneously reported treatment-emergent adverse events in the locked database for each PDSS patient after the PDSS event and up to time of study discontinuation or database lock.

## Results

### Exposure and PDSS incidence

Through 14 October 2008, a total of 30 PDSS events resulting from olanzapine LAI had been reported in 29 patients. Based on approximately 45,000 injections of olanzapine LAI given to 2054 patients in clinical trials as of that date, PDSS events had occurred in approximately 0.07% of injections, or 1.4% of patients. Based on the locked database occurring April 2008, which included 29 PDSS events, 2054 patients had received a total of 41,193 injections of olanzapine LAI with a mean exposure of 14 months (range 2 weeks to 3.8 years) or 20 injections per patient (range 1 to 100 injections), for a total of 2353.5 patient-years of exposure, and yielding a PDSS rate of 1.2 events per 100 patient-years of exposure.

### Description of cases

Case information for each of the 30 PDSS events is summarized in Additional File 1. Events occurred at various doses and at various cumulative exposure lengths. Median injection number at which an event occurred was the 21st injection.

### Symptoms

Table 1 summarizes clinical symptoms occurring in at least two cases and presents the symptoms that occurred initially in the course of the event versus at any time during the event. The most common signs and symptom types were related to delirium and sedation. Delirium-related adverse events, such as disorientation, confusion, ataxia, and dysarthria, were reported in 97% of the events. Sedation-related adverse events, defined here as somnolence, sedation, or other change in level of consciousness, were reported in 87% of the events. All 30 cases presented with at least one symptom related to either delirium or sedation, with 83% of cases resulting in both delirium- and sedation-related symptoms. Initial symptoms (i.e., symptoms first noted by the patient, investigator, or other witness at the onset of the event) included delirium-related symptoms in 47% of cases and sedation-related symptoms in 40% of cases. However, in another 40% of cases, the first symptoms noted did not include signs of sedation or delirium but were instead related to general malaise or other symptoms such as extrapyramidal symptoms (EPS), agitation, anxiety, or irritability. In those cases, the delirium or sedation developed subsequent to the initial symptoms.

**Table 1:** Incidence and timing of signs and symptoms of post-injection delirium/sedation syndrome (PDSS) occurring with olanzapine long-acting injection

Clinical Symptoms of PDSS Events--Grouped	Presented Initially N (%)	Occurred at Any Time N (%)
Sedation (e.g., somnolence, sedation, unconsciousness)	12 (40)	26 (87)
Delirium (combined)	14 (47)	29 (97)
Speech Impairment (e.g., dysarthria)	7 (23)	21 (70)
Motor Impairment (e.g., ataxia)	7 (23)	12 (40)
Cognitive Impairment (e.g., confusion, disorientation)	8 (27)	17 (57)
EPS, akathisia, tension or cramps in extremities	3 (10)	7 (23)
Agitation, aggression, irritability, anxiety, restlessness^a^	2 (7)	9 (30)
General malaise (e.g., weak, dizzy, felt bad)	19 (63)	20 (67)
Hypertension	1 (3)	2 (7)
Possible seizure/convulsions	0 (0)	2 (7)

Abbreviations: e.g. = for example; EPS = extrapyramidal symptoms; N = number of patients; PDSS = post-injection delirium/sedation syndrome.

^a ^Restlessness may also be a manifestation of EPS (akathisia).

### Onset and progression

**Figure 1:**
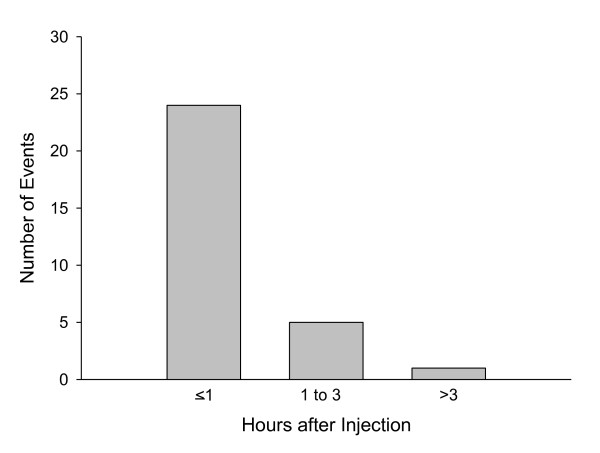
**Approximate onset time of post-injection delirium/sedation syndrome events**.

Time of onset of symptoms for PDSS events ranged from 0 to 300 minutes post injection, with a mean of 49 minutes and a median of 25 minutes post injection. Onset of the events was predominantly clustered in the 1-hour post-injection time frame, with 80% of PDSS events occurring within 1 hour post injection (Figure 1).

**Figure 2:**
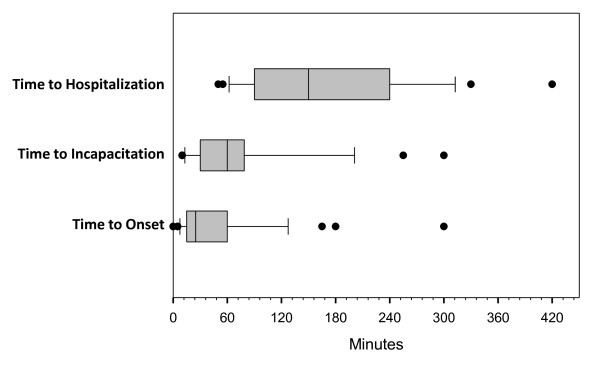
**Post-injection delirium/sedation syndrome events and time to initial onset, incapacitation, and hospitalization**. The middle line inside the box is the median 50th percentile; left border of the box is the 25th percentile and right borders of the box is the 75th percentile; left whisker is the 10th percentile and right whisker is the 90th percentile.

Of the 22 cases that met criteria for incapacitation, 21 cases contained enough information to document when the patient was first observed in an incapacitated state, with incapacitation defined as the presence of clinically significant disorientation, ataxia, or sedation such that the patient would not have been able to seek assistance on his own. Time from injection to time of first observed incapacitation ranged from 10 to 300 minutes post injection, with a mean time of 75 minutes and a median time of 60 minutes. Thus, median time of incapacitation was 35 minutes later than median time of onset (Figure 2).

Of the 23 events in which the patient was sent to the emergency department or hospital, the exact time of hospitalization was documented in 17 cases. Time to hospitalization ranged from 50 to 420 minutes post injection, with a mean of 178 minutes and a median of 150 minutes. Therefore, median time to hospitalization occurred approximately 2 hours after median time of onset of the event (Figure 2).

### Clinical assessments

#### Vital signs

Information about vital signs was available for 26 cases. No clinically significant decreases in vital signs were observed, with no instances of orthostatic hypotension, bradycardia, or respiratory depression. Two patients had clinically significantly increased blood pressure during the event (Case 24 and Case 27; peaks of 210/110 and 160/100, respectively) which subsequently responded to treatment with antihypertensives. Although there were other cases in which the patient experienced increases in blood pressure and/or heart rate during the event, these changes in vital signs were not considered clinically significant and were not sustained throughout the event.

#### Electrocardiograms (ECGs)

Readings of ECGs were obtained for 13 of the 30 PDSS events. In one of these events (Case 26), right bundle branch block was identified. This finding was determined to have resulted from long-standing arterial hypertension and was not related to the administration of olanzapine LAI. No other clinically significant ECG changes were observed for these events.

#### Electroencephalograms (EEGs)

EEGs were obtained in 4 of the 30 PDSS events (Cases 3, 9, 14, and 28). Cases 3 and 28 had clinical presentations described by witnesses as convulsive movements, but the EEG findings did not provide evidence of seizure in either case. The EEG findings for Cases 3 and 14 were reported as normal. In Case 9, which involved a patient with diabetes, the EEG showed a generalized slowing of waves corresponding with metabolic and/or pharmacological encephalopathy. In Case 28, an EEG performed approximately 10 days after the event was reported to be abnormal due to the presence of diffuse disorganization, consistent with findings seen in other patients with schizophrenia, but which was not supportive of paroxysmal activity or epileptic foci and did not indicate the presence of characteristic electrographic seizure activity.

#### Other testing

A computed tomography scan was performed in Cases 2, 3, 5, 9, 13, 14, 24, 28, and 30. In each case, the results of the computed tomography were negative for any clinically significant findings. A urine and/or blood toxicology screen was performed in Cases 3, 4, 5, 9, 10, 13, 14, 25, and 30. In one case (Case 3), the patient was treated with benzodiazepines upon arriving at the hospital, resulting in a blood toxicology screen that was positive for benzodiazepines. In all other cases, the urine and/or blood toxicology screens were negative for alcohol, sedatives, or illicit substances.

### Hospitalization and treatment

In 77% of cases (23/30), the patient was sent to the hospital at some time during the PDSS event. The majority (63%) of PDSS events resolved either with no treatment or were managed with only observation and fluids. In 11 of the 30 PDSS events, the patient was hospitalized and received medical treatment beyond observation and fluids. In 9 of these 11 events, the patients received various medications while hospitalized. Although these medications were often used to treat specific symptoms or concomitant medical illness (e.g., biperiden for EPS; insulin or glibenclamide for elevated blood glucose; enalapril, captopril, propranolol, or magnesium sulphate for hypertension; antibiotics for infection; benzodiazepines or other tranquilizers for agitation), some (e.g., lucetam and cerebrolysin in one case) appear to have been given prophylactically. Six patients received treatment with benzodiazepines. Three patients were catheterized: one because of urinary retention and two prophylactically. One patient was placed in mechanical restraints because of agitation. Two patients (Cases 3 and 14) were ventilated as a preventative measure following benzodiazepine administration, one due to apparent seizure and the other to manage severe agitation so that a computed tomography scan could be performed; no respiratory depression had been noted in either case.

### Recovery and post-recovery outcomes

**Figure 3:**
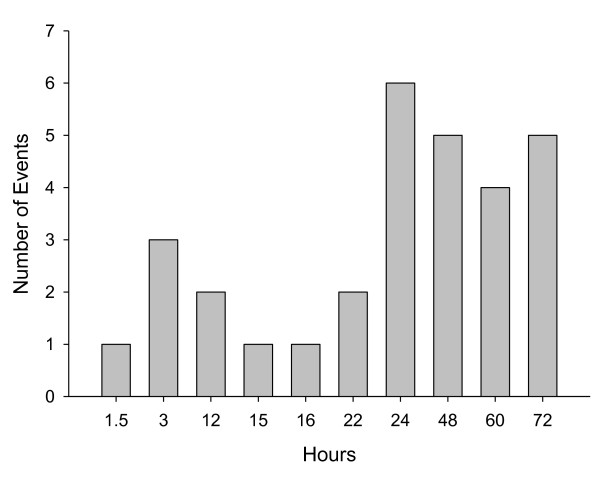
**Approximate time to recovery from post-injection delirium/sedation syndrome events**.

All patients fully recovered from the PDSS event, with recovery occurring from 1.5 to 72 hours after onset (Figure 3). In the majority of cases (21/30; 70%), patients continued to receive further treatment with olanzapine LAI following the event. At the time of datalock, median number of additional days of study participation following a PDSS event was 184 days, and median number of subsequent injections received following the event was 9.

To assess for potential long-term outcomes associated with PDSS events, all adverse events experienced by the patient after the PDSS event and up to time of study discontinuation or database lock were reviewed. Of the 28 patients who experienced a PDSS event prior to the last database lock, 18 patients had no additional adverse events of any kind reported following the PDSS event. For the 10 patients who did have subsequent adverse events reported, a total of 20 adverse events of any kind were reported following the PDSS event. Only one patient experienced a second PDSS event (see Cases 5 and 8), with the second event occurring approximately 6 months after the first event. Otherwise, a review of the subsequent adverse events indicated that, with the exception of one report of "dry mouth" 1 day after the event for one patient, patients who experienced a PDSS event and continued study participation did not subsequently report any events that appeared to be potentially related to the PDSS event. Examples of these unrelated subsequent adverse events included uterine fibromioma (78 days after event), erectile dysfunction (192 days after event), pharingitis (379 days after event), and psychotic exacerbation (659 days after event). In addition, the absolute number of adverse events overall did not appear to increase following a PDSS event compared with prior to the PDSS event. Median number of adverse events experienced by patients prior to the PDSS event was one; median number of adverse events reported by patients at any time after the PDSS event was zero.

### Post-recovery dosing

In 13 cases, there was no change in dose following the PDSS event. In 8 cases, the olanzapine LAI dose was decreased at the next injection following the PDSS event. No patients received oral olanzapine supplementation following the PDSS event.

### Analysis of risk factors

No clear risk factors for PDSS were identified. Of the variables assessed in the logistic regression analysis, only three met criteria to enter and remain in the model (p < 0.20): BMI (p = 0.033), age (p = 0.035), and dose (p = 0.126). However, the odds ratios clustered around 1.0, indicating a small incremental increase in risk for each unit decrease in BMI (odds ratio = 0.92 [95% CI 0.86-0.99] and for each year increase in age (odds ratio = 1.03 [95% CI 1.00-1.07], but no significant increase in risk based on dose (odds ratio = 1.00 [95% CI 1.00-1.01]).

No concomitant medications were identified as risk factors. There was no class of drugs with statistically significantly higher incidence of use among patients experiencing PDSS compared with patients not experiencing PDSS. Statistically significant differences were noted with respect to three specific medications (escitalopram, p = 0.026; fluvoxamine, p = 0.040; oxaprozin, p = 0.014); however, those findings appeared driven by the very small numbers of patients using those medications in the total sample (N's of 19, 3, and 1, respectively) and are likely spurious.

## Discussion

### Incidence

During clinical trials of olanzapine LAI, 30 post-injection delirium/sedation syndrome cases occurred in 29 patients from August 2000 to October 2008. The incidence of PDSS events was 0.07% of injections or approximately one event per 1400 injections. To put this observed rate into clinical context, a clinic with 60 patients receiving an injection every 2 weeks might expect approximately 1 event per year; a clinic with 60 patients receiving an injection every 4 weeks might expect 1 event every 2 years.

### Symptoms and identification of cases

Symptoms of PDSS were consistent with some of those in oral olanzapine overdose, including dizziness, confusion, disorientation, slurred speech, altered gait, weakness, muscle spasms, possible seizure, and varying degrees of sedation. It should be noted that not all of the symptoms reported with oral olanzapine overdose were seen in patients during PDSS events. For example, neither orthostatic hypotension nor respiratory depression were reported. In many instances, the clinical picture of PDSS was described as similar to that of alcohol intoxication. Overall, the syndrome is best characterized and identified by a convergence of symptoms related to transient delirium and/or excessive sedation. Based on these findings from the clinical trials, a working case definition is proposed in Appendix A.

Not all of the cases initially began with symptoms of sedation or delirium but instead began with nonspecific symptoms of malaise (e.g., dizziness, weakness, not feeling well). Therefore, it will be important for healthcare providers and patients to be alert for the development of any of these potential early warning signs in order to identify PDSS events at the earliest possible time to ensure that appropriate measures are taken to maintain the patient's safety.

### Onset and progression

Data regarding time of onset indicated that the risk of occurrence of a PDSS event was greatest within the first hour, although events also occurred rarely (< 1 in 1,000 injections) between 1 and 3 hours and very rarely (< 1 in 10,000 injections) after 3 hours. Although PDSS events can be serious in nature, events have not been characterized by a sudden onset of incapacitation but usually began with milder symptoms that then progressed in number and/or severity. This gradual onset thus allows time for those around the patient to identify the syndrome and respond accordingly.

The duration of the PDSS event has varied. Although milder events took from 1.5 to 3 hours to resolve, most events required about 24 to 72 hours to resolve fully. For the 7 patients who lost consciousness at any time, the longest period of unconsciousness was 12 hours.

### Treatment and outcomes

Although most patients were hospitalized during the event, a majority of PDSS events either resolved with no treatment or were managed with only observation and fluids. When active treatments were given, this tended to be in response to the development of specific symptoms--either related or unrelated to PDSS, such as EPS, elevated blood glucose, hypertension, infection, or agitation. The majority of patients who experienced a PDSS event continued to receive further injections of olanzapine LAI. Those who continued to participate in the studies did not require oral antipsychotic supplementation following the event and were able to receive their next injections of olanzapine LAI at their next regularly scheduled injection visits. A review of adverse events experienced by patients in the days, months, and years after the PDSS event supports the conclusion that patients recovered from the event with no lingering or permanent sequelae.

### Risk factors

No concomitant medications or precipitating events could be identified which might predispose a patient toward the occurrence of PDSS. However, there appears to be limited evidence suggesting that individual patient factors related to patients' general health and physical status could incrementally increase the risk of an event. Regression analyses indicated that lower BMI and/or higher age could increase risk somewhat. Counterbalancing these statistical findings, it is important to note that PDSS events occurred in patients at many different ages and BMIs. Therefore, younger age and higher BMI do not prevent the occurrence of PDSS, nor do higher age and lower BMI necessarily predict its occurrence. Instead, PDSS can potentially occur at any injection in any patient, suggesting the need to monitor all patients for its possible occurrence.

As discussed by McDonnell et al. [5], the probable mechanism most likely involves accidental entry of the medication into the blood stream following blood vessel injury during the injection process. The similarity in incidence of olanzapine LAI PDSS (0.07% of injections) to that of Hoigne's syndrome following accidental intravascular injection of penicillin procaine G (0.08% of injections) [6] suggests that these findings may be approximating the naturally occurring background rate for accidental direct or indirect intravascular injection during any intramuscular injection process.

Nevertheless, another finding which suggests that there may also be individual patient factors which could affect the occurrence of PDSS is the fact that one patient in the clinical trials experienced this event twice. Examination of medical history and concomitant medications indicated that this patient had a number of chronic conditions (including diabetes, arthritis, alcoholism, and hypertension) and may have been in poorer general health than the other PDSS patients, although such conditions are not at all uncommon in patients with schizophrenia and are certainly represented among the patients who did not experience PDSS. It is important to note that McDonnell et al. [5] found olanzapine pamoate to be significantly more soluble in blood, such that accidental contact of the medication with a large quantity of blood may produce higher than expected olanzapine plasma concentrations in a manner that would be consistent with the clinical presentation and timing of the symptoms of PDSS. Thus, a patient who was more prone to bleeding and/or vessel injury might theoretically be at greater risk for this accidental occurrence. Chronic salicylate usage has been associated with increased clotting time and risk of bleeding [20-22], as has alcoholism [23,24]. Also, chronic diabetes can result in vascular fragility [25,26]. Thus, any or all of these factors may have created a predisposition toward excessive bleeding and/or increased likelihood of vessel injury in this patient. Beyond the idiosyncratic situation with this particular patient, the hypotheses generated by this case may converge with the statistical findings of greater age and lower BMI as weak risk factors for PDSS if one assumes that greater age is associated with a higher likelihood of poorer general health and likelihood of vascular fragility and if lower BMI is associated with higher likelihood of vascular injury during the injection. However, none of these hypotheses have been able to be confirmed. Further research on a larger number of cases is needed to explore more fully the possibility of specific risk factors.

### Risk management

Because PDSS events likely involve a portion of the olanzapine LAI dose coming into contact with blood, and because accidental intravascular administration is a known risk of intramuscularly administered drugs, use of appropriate injection technique is a necessary precaution. The injection administrator should aspirate for several seconds to ensure that no blood appears before injecting the medication. If any blood is aspirated into the syringe, the clinician should discard the syringe, reconstitute a new vial of olanzapine LAI, and inject the medication into the alternate buttock deep into the gluteal muscle. Nursing texts and scientific literature also state that the ventrogluteal location is the preferred injection site for intramuscular injections [27,28]. The rationale for this recommendation is that the ventrogluteal site is less likely to access the sciatic nerve or a major blood vessel; this site also maximizes the likelihood of achieving an intramuscular injection, as opposed to an accidental subcutaneous injection.

Because proper injection technique does not guarantee that a blood vessel injury has not occurred during the injection process, it is therefore necessary to observe the patient following each injection. Following each injection of olanzapine LAI, healthcare personnel should observe the patient at the healthcare facility for at least 3 hours for signs and symptoms consistent with olanzapine overdose and should confirm that the patient is alert, oriented, and absent of any signs and symptoms of overdose prior to discharge. Because of the risk that a PDSS event could still occur following this 3-hour period, patients will need to have someone go with them to their destination upon leaving the facility after the observation period. They should also be advised to be vigilant for symptoms of post-injection adverse reactions, should be able to obtain medical assistance if needed, and should not drive or operate heavy machinery for the remainder of the day of the injection. These precautions are summarized in Appendix B. Note that if a PDSS event is suspected, close monitoring and supervision of the patient should continue until examination indicates that signs and symptoms have resolved. Also, if parenteral benzodiazepines are required for the management of post-injection adverse reactions, careful evaluation of clinical status for excessive sedation and cardiorespiratory depression are recommended.

## Conclusion

In conclusion, post-injection delirium/sedation syndrome has occurred following 0.07% of injections of olanzapine LAI, with 1.2 events occurring per 100 patient years of exposure. These events are readily identifiable and have been successfully managed during the clinical trials. All patients have recovered with no lingering or permanent sequelae, and most have elected to continue receiving treatment with olanzapine LAI and have not had additional PDSS events. However, because of the need for a 3-hour post-injection observation period as well as additional precautions for the remainder of the day of the injection, it will be important for clinicians and their patients to weigh the risks and benefits of treatment with olanzapine LAI and to determine ahead of time whether the patient will be able to make arrangements in order to comply with the necessary precautions.

## Competing interests

All authors are employees of Eli Lilly and Company.

## Authors' contributions

HD- study design, data collection, analysis and interpretation, and drafting of the manuscript; DM- study design, data collection, analysis, and interpretation; EB- data collection, analysis, and interpretation; FZ- statistical analyses and interpretation; SS- data collection, analysis, and interpretation, literature review; VS- analysis and interpretation of data, assistance with drafting of the manuscript; SC- analysis and interpretation of data. All authors read and approved the final manuscript.

## Appendix A

### Proposal for Working Case Definition of Olanzapine Long-Acting Injection Post-Injection Delirium/Sedation Syndrome (PDSS)

Criteria 1 through 4 must be met for a clinical diagnosis of post-injection delirium/sedation syndrome.

1. One or both of the conditions listed in (a) and (b):

a. A minimum of 1 sign or symptom from at least 3 of the following symptom clusters consistent with olanzapine* overdose with one or more of at least moderate severity.

i. Sedation/somnolence

ii. Delirium/confusion/disorientation/other cognitive impairment

iii. Dysarthria/other speech impairment

iv. Ataxia/other motor impairment

v. Extrapyramidal symptoms

vi. Agitation/irritability/anxiety/restlessness

vii. Dizziness/weakness/general malaise

viii. Seizure

b. Any one of the following signs and symptoms such that patient is: 

unarousable

unconscious

stuporous

comatose

* Other signs and symptoms not listed under 1a may occur with olanzapine overdose but are not considered criteria for PDSS. See olanzapine prescribing information.

2. Condition develops within 24 hours of an olanzapine long-acting injection.

3. Condition cannot be explained by a significant dose increase of olanzapine long-acting injection, initiation or addition of oral olanzapine or other sedating medication, or new exposure to olanzapine long-acting injection.

4. Underlying medical conditions have been ruled out, including concomitant substance use or abuse.

## Appendix B

### Safety Precautions for Each Administration of Olanzapine Long-Acting Injection

#### Before the Injection

• Determine that the patient will not travel alone to their post-injection destination

#### During the Injection

• Aspirate the syringe for several seconds following insertion of the needle into the muscle to ensure that no blood appears before injecting the medication. If any blood is aspirated into the syringe, discard the syringe, reconstitute a new vial of olanzapine LAI, and inject into the alternate side of the buttock.

#### After the Injection

• Patients should be observed in a healthcare facility by appropriately qualified personnel for at least 3 hours for signs and symptoms consistent with olanzapine overdose.

#### Before Leaving the Healthcare Facility

• Confirm the patient is alert, oriented, and without signs or symptoms of post-injection delirium/sedation syndrome (PDSS) event.*

• Advise patients to be vigilant for symptoms of a PDSS event for the remainder of the day and be able to obtain medical assistance if needed.

#### After Leaving the Healthcare Facility

• Patients should not drive or operate machinery for the remainder of the day.

* If post-injection delirium/sedation syndrome is suspected, close medical supervision and monitoring should continue until examination indicates that signs and symptoms have resolved. If parenteral benzodiazepines are required for the management of post-injection adverse events, careful evaluation of clinical status for excessive sedation and cardiorespiratory depression is recommended.

## Pre-publication history

The pre-publication history for this paper can be accessed here:

http://www.biomedcentral.com/1471-244X/10/43/prepub

## Supplementary Material

Additional file 1 **Descriptions of the first 30 cases of olanzapine long-acting injection postjection delirium/sedation syndrome**. This file is a table presenting patients' age, gender, dose, concomitant medications, timing of PDSS onset, injection number, whether the patient was hospitalized during the event, treatment received during the event, narratives of the events, time to recovery, and subsequent disposition of the patients in the clinical trials. Click here for file
