# Supplementary material for: Post-injection delirium/sedation syndrome in patients with schizophrenia treated with olanzapine long-acting injection, I: analysis of cases
Source: BMC Psychiatry. 2010 Jun 10;10:43. doi: 10.1186/1471-244X-10-43 (PMC2895589; doi:10.1186/1471-244X-10-43)
Supplement: Additional file 1 — Descriptions of the first 30 cases of olanzapine long-acting injection postjection delirium/sedation syndrome. This file is a table presenting patients' age, gender, dose, concomitant medications, timing of PDSS onset, injection number, whether the patient was hospitalized during the event, treatment received during the event, narratives of the events, time to recovery, and subsequent disposition of the patients in the clinical trials. [file 1471-244X-10-43-S1.PDF]

Additional file 1. **Descriptions of the first 30 cases of olanzapine long-acting injection postjection delirium/sedation syndrome.**

Case #	Age, sex	Dose	Concomitant Medications	Onset, Inj #	Hospital-ization	Treatment	Description, duration, disposition
**1**	31/M	300mg/ 4wks	None	45 min/ Inj #2	No	Biperiden	45 min post inj, experienced severe sedation, moderate tension (akathisia) in legs, mild dizziness, weakness. Disoriented, spoke briefly, fell asleep. 6 hrs after inj, still sleepy but felt better. **Recovered approx 48 hr; continued in study**
**2**	32/M	405mg/ 4wks	None	10 min/ Inj #1	Yes	Fluids, mannitol, lucetam (priacetam), cerebrolysin, glucose, infesol	10 min post inj, experienced dizziness & bad general state. Speech progressively altered & somnolence appeared. After 1.5 hr, stopped responding to verbal stimuli. After 2 hr, profound sedation, bilateral miosis with no photomotor reflex, automatic movements, Babinski on left side, no response to pain or verbal stimuli. Tests negative. Able to speak next morning but with difficulty. **Recovered approx 60 hr; discontinued study**
**3**	63/M	405mg/ 4wks	None	15-20 min/ Inj #2	Yes	Midazolam, ranitidine diazepam, haloperidol, promethazine; ventilated	15-20 min post inj, appeared pale, yellowish, not standing steady, a little confused. 30 min post inj, felt bad, disoriented, with "seizures in hands & legs" which appeared as clonic movements of limbs without loss of consciousness. Disoriented with psychomotor agitation. Ventilated as preventive measure after benzodiazepines. **Recovered approx 60 hr; discontinued drug; continued into oral olanzapine extension period of study**
**4**	30/M	405mg/ 4wks	None	~60 min/ Inj #4	Yes	Unknown	Appears to have presented self at hospital. Approx 1 hr post inj, experienced sedation. Became drowsy, irritable, disoriented x 3. Felt stiff & weak in legs. Stated that he passed out for a while, was very confused, slightly febrile (100.6 degrees F) **Recovered approx 48 hr; continued in study**
**5**	50/M	250mg/ 2wks	Glipizide, amlodipine, ranitidine, $CaCO_3$, colchicine, aspirin, lisinopril, oxaprozin	Within 60 min/ Inj #22	Yes	None	Returned to site about 1 hr post inj; appeared in drunken state. Speech was slurred, gait unsteady. Sent to hospital; all test neg. Difficulty ambulating, incontinent of urine while at hospital. Reported drinking ¾ pint of whiskey the evening before the inj. **Recovered approx 48 hr; continued in study**

Continued

<u>Additional file 1.</u> Descriptions of the first 30 cases of olanzapine long-acting injection postjection delirium/sedation syndrome.

**6**	52/M	300mg/ 2wks	None	Within 50 min/ Inj #24	Yes	Fluids	Pt left site 10 min post inj. Was found in "coma" approx 50 min later. According to investigator, pt was riding on a bus when he began to feel unwell. He got off the bus and was later found on a bench in a public plaza. Pt was hospitalized; remained unconscious & unresponsive to verbal stimuli for approx 12 hr. **Recovered approx 22 hr; continued in study**
**7**	32/F	300mg/ 3wks	Amitriptyline, oral olanzapine	30 min/ Inj #11	Yes	None	30 min post inj, experienced drowsiness and "washy speech." Admitted to psych hospital. Also experienced slight confusion (nonserious). **Recovered approx 24hr; continued in study**
**8**	50/M	250mg/ 2wks	See Case 5.	15 min/ Inj #35	Yes	None	15 min post inj, began to have slurred speech & unsteady gait. Progressed to point where could not speak clearly or ambulate without assistance. Taken to hospital for evaluation. Tests neg. This was the second event for this patient (see Case 5). **Recovered approx 72 hr; discontinued study**
**9**	34/M	300mg/ 4wks	None	5 min/ Inj #29	Yes	Insulin, haloperidol, omeprazole, alprazolam, fluids	Pt diabetic. 5min post inj, became increasingly sedated. In & out of consciousness. Site assumed low glucose & gave pt Coke to drink. Pt confused, ataxic (as if drunk). 30 min post inj, glucose was 275 mg/dL. Site laid pt down in ward, where he was in & out of sleeping state. When would try to get up, was restless & had slurred speech. Next day pt was still sleepy & disoriented, delirious, with slight rigidity in extremities, high glucose with slight hypokalemia. Tests indicated hepatic steatosis. **Recovered approx 72 hr; continued in study**
**10**	45/M	405mg/ 4wks	None	30 min/ Inj #20	Yes	None	Pt returned to work soon after inj. Within 30 min post inj, felt bad. Approx 60 min post inj, pt noted to have somnolence, dysarthria, irritability. Coworkers contacted site & returned pt to site. Pt had difficulty walking, became sedated. Sent to hospital for observation. **Recovered approx 24 hr; continued in study**
**11**	45/F	100mg/ 2wks	Diazepam, escitalopram	10 min/ Inj #27	Yes	None	10 min post inj, pt experienced weakness, dizziness, slurred speech, profound sedation (described as slightly decreased level of consciousness). **Recovered approx 48 hr; continued study**
**12**	58/M	210mg/ 2wks	None	Unspec-ified. Within 3 hrs. / Inj #2	No	None	3 hr post inj, felt weak. Wife contacted site, reported that pt was experiencing profound sedation, weakness, slurred speech. Not unconscious. Remained at home. **Recovered approx 3 hr; continued in study**

Continued

Additional file 1. **Descriptions of the first 30 cases of olanzapine long-acting injection postjection delirium/sedation syndrome.**

**13**	25/M	270mg/ 4wks	None	Immed-iately post inj/ Inj #18	Yes	None	Immediately post inj, pt complained of weakness, dizziness, headache. Stated that he'd been working outside all day in warm weather without eating or drinking. Stayed at site 45 min but then left per investigator instructions to get something to eat. Pt got sandwich on street; felt unwell as started to eat. Began staggering; attempted to go into bar but was turned away as appeared drunk. Shopkeeper called emergency services. 3 hr post inj, admitted to hospital consfused & dizzy. All tests neg. **Recovered approx 21 hr; continued in study**
**14**	58/F	210mg/ 4wks	Fenofibrate	Unspecified.Within 75 min/ Inj #25	Yes	IV midazolam, sufentanil, enoxaparine, furosemide, fluids; ventilated	Pt refused to stay at site; left 20-25 min post inj. Experienced malaise 75 min post inj; admitted to hospital with loss of consciousness. Experienced agitation, somnolence, dysarthria, sweating, mild tachycardia (114 bpm). Due to persistence of agitation, given IV midazolam & intubated & ventilated to perform tests. Pt extubated & released. **Recovered approx 60 hr; continued in study**
**15**	41/M	300mg/ 3wks	Lorazepam	15 min/ Inj #7	Yes	Urinary catheterization	15 min post inj, became confused & weak. 1 hr 30 min post inj, condition worsened; pt appeared stunned, with deep sedation, loss of consciousness. **Recovered approx 3 hr; discontinued study**
**16**	37/M	405mg/ 4wks	None	75 to 105 min/ Inj #17	Yes	None	75-105 min post inj, experienced somnolence. 60-90 min later, experienced fatigue, inconsistent speech, mumbling, & automatism (picking up invisible things on the floor– pseudo-delirium). Hospitalized overnight for confusional state. Pt later reported drinking 1 liter of beer prior to inj. **Recovered approx 24 hr; continued study**
**17**	61/F	300mg/ 2wks	Clonazepam, trihexy-phenidyl	2 hr 45 min/ Inj #27	Yes	None	2 hr 45 min post inj, experienced significant somnolence. Pt took 4 mg unprescribed clonazepam 8 hr prior to inj (but did not appear drowsy when arrived at site). Approx 3 hr post inj, experienced difficulty in speech but still alert & oriented; displayed motor restlessness. 6-7 hr post inj, presented with profound sedation; unarousable for 8 hr. Responsive to pain. Awoke next morning. **Recovered approx 15 hr; continued in study**
**18**	27/M	345mg/ 4wks	Oral olanzapine	30 min/ Inj #17	Yes	Fluids	30 min post inj, experienced dizziness, "gummy legs," insecurity while standing. Symptoms slowly increased, progressing to deep sedation, reported to be like deep sleep but pt could always be aroused by speaking to him loudly. Hospitalized for monitoring & hydration. **Recovered approx 24 hr; discontinued study**

Continued

Additional file 1. Descriptions of the first 30 cases of olanzapine long-acting injection postjection delirium/sedation syndrome.

**19**	39/F	390mg/ 4wks	Fluoxetine	5 min/ Inj #16	No	None	5 min post inj, experienced somnolence that worsened gradually; pt was oriented & able to communicate although had dysarthria. Event was described as nonserious by investigator. At end of 3-hr observation, pt was sent home with a friend in an improved but still slightly somnolent state. **Recovered approx 72 hr; discontinued study**
**20**	50/F	405mg/ 4wks	Estazolam, clorazepate dipotassium, iron, folic acid	20 min/ Inj #15	No	None	20 min post inj, experienced dizziness. 45 min post inj, was severely sedated but always conscious; was disoriented to place & time, with dysarthria & confusion. Site was attached to psych unit where pt lived for social reasons; pt was able to be observed by staff there until recovered. **Recovered approx 16 hr; continued in study**
**21**	52/M	210mg/ 2wks	Lorazepam	15 min/ Inj #35	Yes	Fluids	15 min post inj, became confused, somnolent, with blurred vision, dizziness. All events considered nonserious. 2.5 hr post inj, sent to hospital for monitoring. Remained conscious throughout. **Recovered approx 12 hr; continued in study**
**22**	52/M	360mg/ 4wks	Fluvoxamine	10 min/ Inj #20	Yes	Urinary catheterization	10 min post inj, became somnolent, confused, developed cramps. Slept for 30 min. Arousable but couldn’t answer questions correctly. Disoriented with altered consciousness but not unconscious. Experienced retention of urine. Sent to hospital after 4 hrs of observation. Pt did not urinate despite attempts, so was catheterized. Cramps of moderate severity localized in arms & legs. **Recovered approx 24 hr; discontinued study**
**23**	47/M	405mg/ 3wks	Lorazepam, disulfiram	Within 30 min/ Inj #17	Yes	None	Pt complained of dizziness prior to inj, probably due to fasting. Symptoms worsened. Pt ate 15-30 min post inj; while eating began to feel nervous & experienced abnormal movements like tonic convulsion in arms, sporadic at first & then increasing. 2 hr post inj, began to present somnolence & dysarthria but nervous & with abnormal movements so unable to fall asleep. Given 1 mg lorazepam (his usual daily dose). No loss of consciousness at any time. Sent to hospital at 4 hr post inj due to continued symptoms. **Recovered approx 24 hr; discontinued study**
**24**	55/M	330mg/ 4wks	None	30 min/ Inj #40	Yes	Captopril, enalapril, paracetamol, IV antibiotics, IV diazepam	Pt’s medical history revealed untreated hypertension for the year prior to the event. BP prior to inj was 140/90 mmHg. 30 min post inj, increased to 180/90. 45 min post inj, complained of headache & stomachache. 60 min post inj, was confused, ataxic, restless. Highest BP was 210/110, managed & resolved with enalapril & captopril. Because of restless, given IV diazepam; slept. Diagnosed with UTI. **Recovered approx 60 hr; discontinued study**

Continued

Additional file 1. Descriptions of the first 30 cases of olanzapine long-acting injection postjection delirium/sedation syndrome.

**25**	36/M	405mg/ 4wks	None	15 min/ Inj #36	Yes	IV fluids	15 min post inj, started experiencing dizziness, dysarthria, gait disturbance. With progressive deepening of sedation over the next 10 min. Pt sent to emergency room 6 hr 40 min post inj, where remained sedated, disoriented, confused. **Recovered approx 48 hr; continued in study**
**26**	73/F	195mg/ 2wks	Lorazepam, verapamil, multivitamin, cod liver oil, benzatropine, furosemide, escitalopram, triamcinolone, estrogren/ progesterone, simethicone	11 min/ Inj #68	No	None	Approx 11 min post inj, complained of generalized weakness, palpitations, heaviness sensation in head. ECG showed a right bundle branch block secondary to long-standing arterial hypertension. Pt gradually presented with sedation, slurred speech, & somnolence approx 30-45 min post inj. Was observed for 7 hr post inj and discharged in stable condition. **Recovered approx 12 hr; continued in study**
**27**	28/M	300mg/ 2wks	None	60 min/ Inj #66	No	MgSO4	Pt reported feeling “constrained” just after inj. 60 min post inj became sleepy, confused, disoriented, weak. BP at 60 min post inj recorded at 160/100 & managed with IV magnesium sulfate, with consequent decrease in BP reading. Pt was released from observation same day; reported as recovered by the following day. **Recovered approx 22 hr; continued in study**
**28**	45/M	405mg/ 4wks	None	Not known. Reported at 300 min/ Inj #44	Yes	IV fluids, KCl, MgSO4, IV diazepam, oral propanolol, glibenclamide, lorazepam, IV ranidtidine, aspirin, chloranphenicol optic drops, foley catheter	Pt found on hospital grounds with confusion, ataxia, & apparent remains of vomit approx 5 hr post inj. Approx 5.5 hr post inj, pt experienced apparent tonic-clonic convulsions for 10-15 sec. Admitted to ER for observation. Reported with tachycardia, dehydration, disorientation, confusion, incoherence, altered states of consciousness, with fluctuations of aggressiveness, agitation, & sleepiness. Administered benzodiazepines and IV fluids. EEG normal, CPK increased. Pt had limited recollection of event; discharged 5 days later with diagnosis of delirium. **Recovered approx 72 hr; continued in study**
**29**	45/M	300mg/ 2 wks	Clonazepam, zopiclone	90 min/ Inj #64	No	None	90 min post inj, pt presented with mild to moderate somnolence, dizziness, confused consciousness state, difficulty speaking, weak legs while standing. 3 hr post inj, pt showed full resolution of all events. **Recovered approx 1.5 hr; continued in study**

Continued

Additional file 1. Descriptions of the first 30 cases of olanzapine long-acting injection postjection delirium/sedation syndrome.

**30**	42/F	300mg/ 4 wks	Naproxen	15 min/ Inj #45	Yes	clonazepam, haloperidol	Prior to inj, pt appeared anxious, restless, with possible akathisia. 15 min post inj, experienced increased anxiety, restlessness, which progressed to moderate agitation. Treated with clonazepam and then developed somnolence. 30 min post inj, experienced dizziness, weak legs, appearance of drunken state, dysarthria, difficulty walking, agitation, and mental confusion. 1 hr post inj, pt was unintelligible with increased sedation in combination with restlessness, confusion, delirium-type behavior and ataxia. Sent to ER at 90 min post inj; treated with IM haloperidol for agitation. Significantly improved 24 hrs later. Of note, prior to visit patient took single dose of naproxen due to pain in legs. **Recovered approx 72 hr; continued in study**

Abbreviations: # = number; AE = adverse event; Age= age at time of event; approx = approximately; BP = blood pressure; bpm = beats per minute; $CaCO_3$= calcium carbonate; cont'd = continued; CPK = creatine phosphokinase; ECG = electrocardiogram; EEG = electroencephalogram; ER = emergency room; F = female; hr = hour; IM = intramuscular; inj = injection; IV = intravenous; KCl = potassium chloride; min = minutes; neg = negative; psych = psychiatric; SAE = serious adverse event; M = male; MgS04 = magnesium sulfate; UTI = urinary tract infection; wks = weeks.
